# Supplementary figures and images for: Protection of Photosynthesis by UVR8 and Cryptochromes in Arabidopsis Under Blue and UV Radiation
Source: Plant Cell Environ. 2025 May 11;48(8):6321–35. doi: 10.1111/pce.15608 (PMC12223700; doi:10.1111/pce.15608)

GR

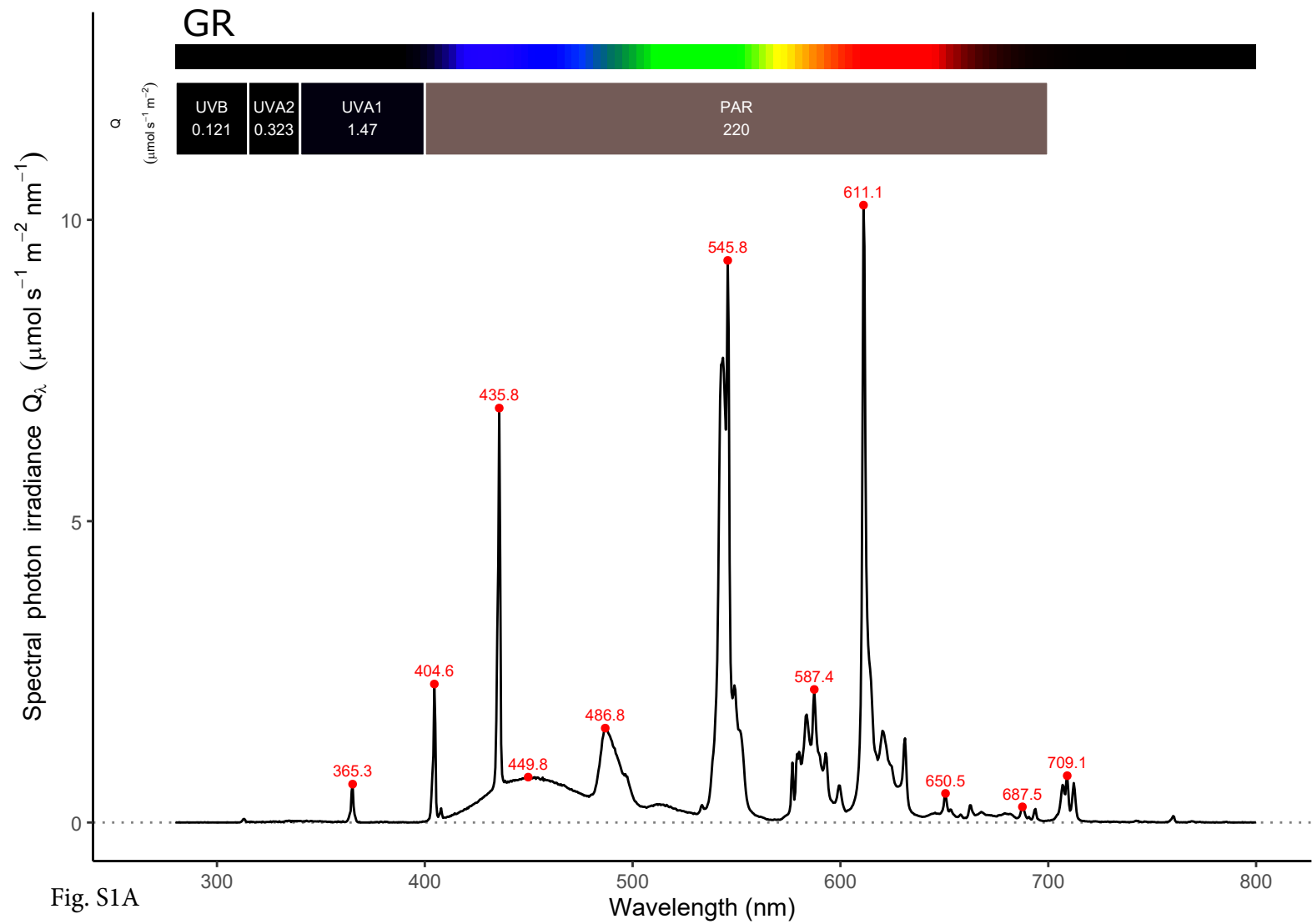

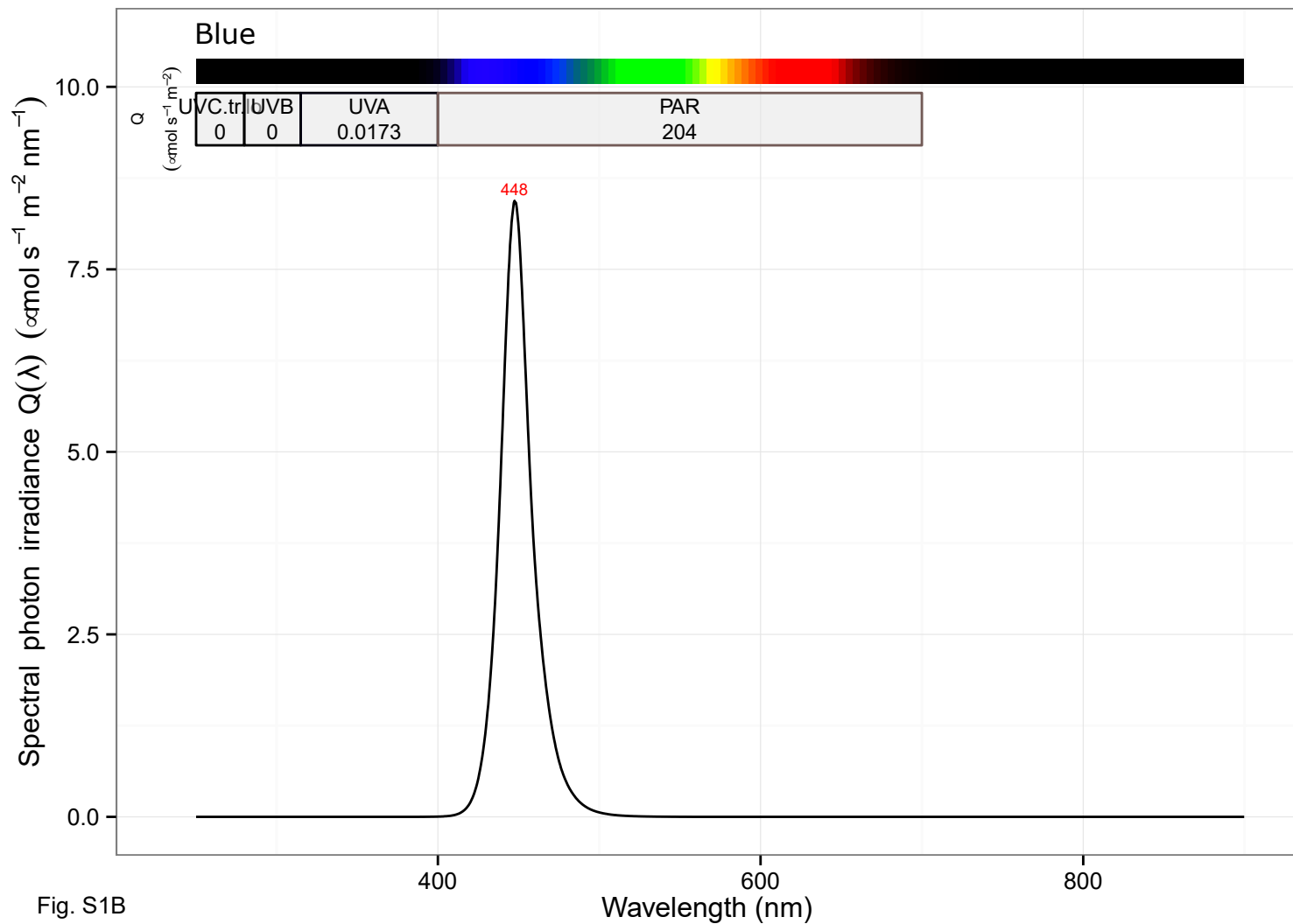

Fig. S1B

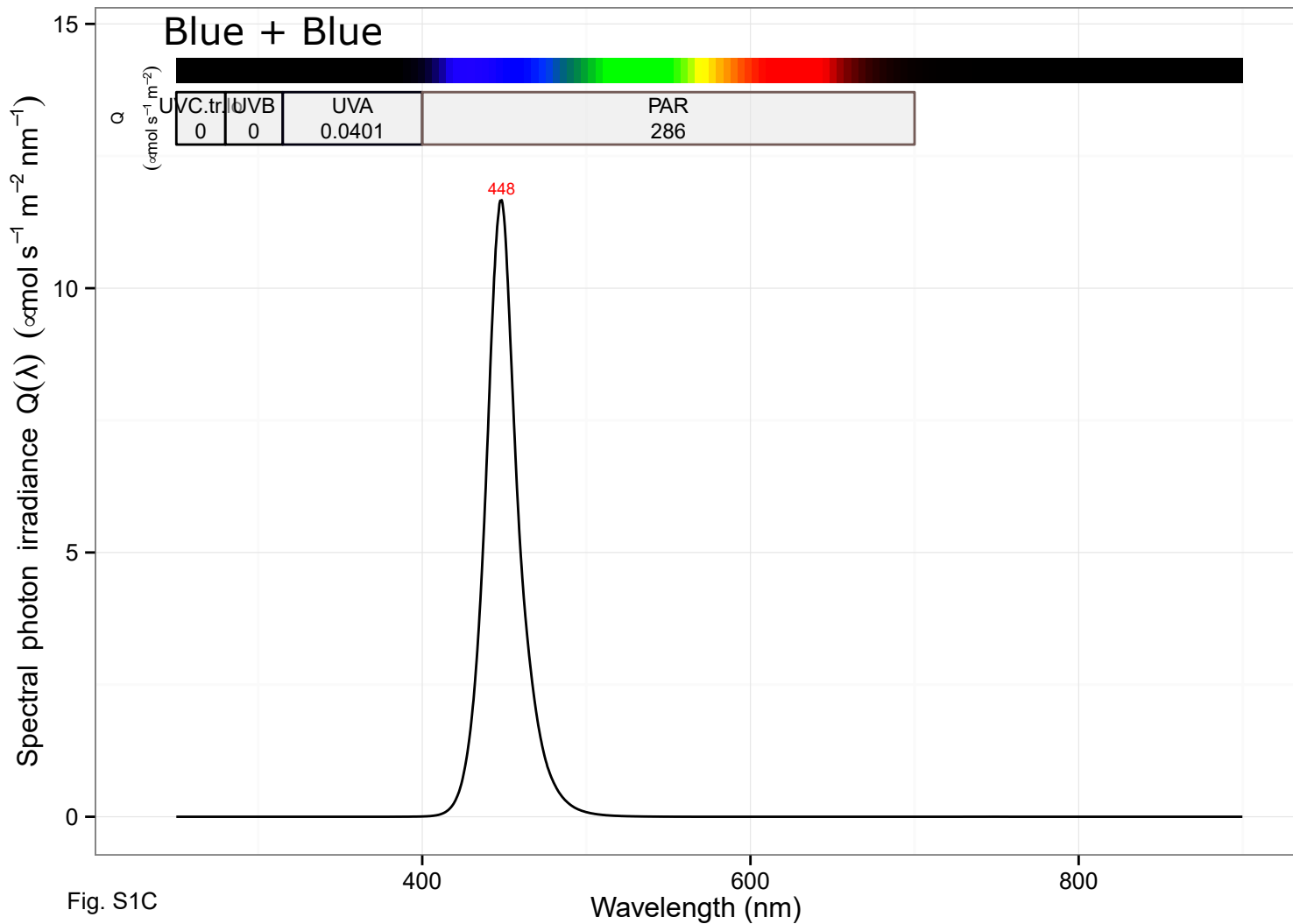

Fig. S1C

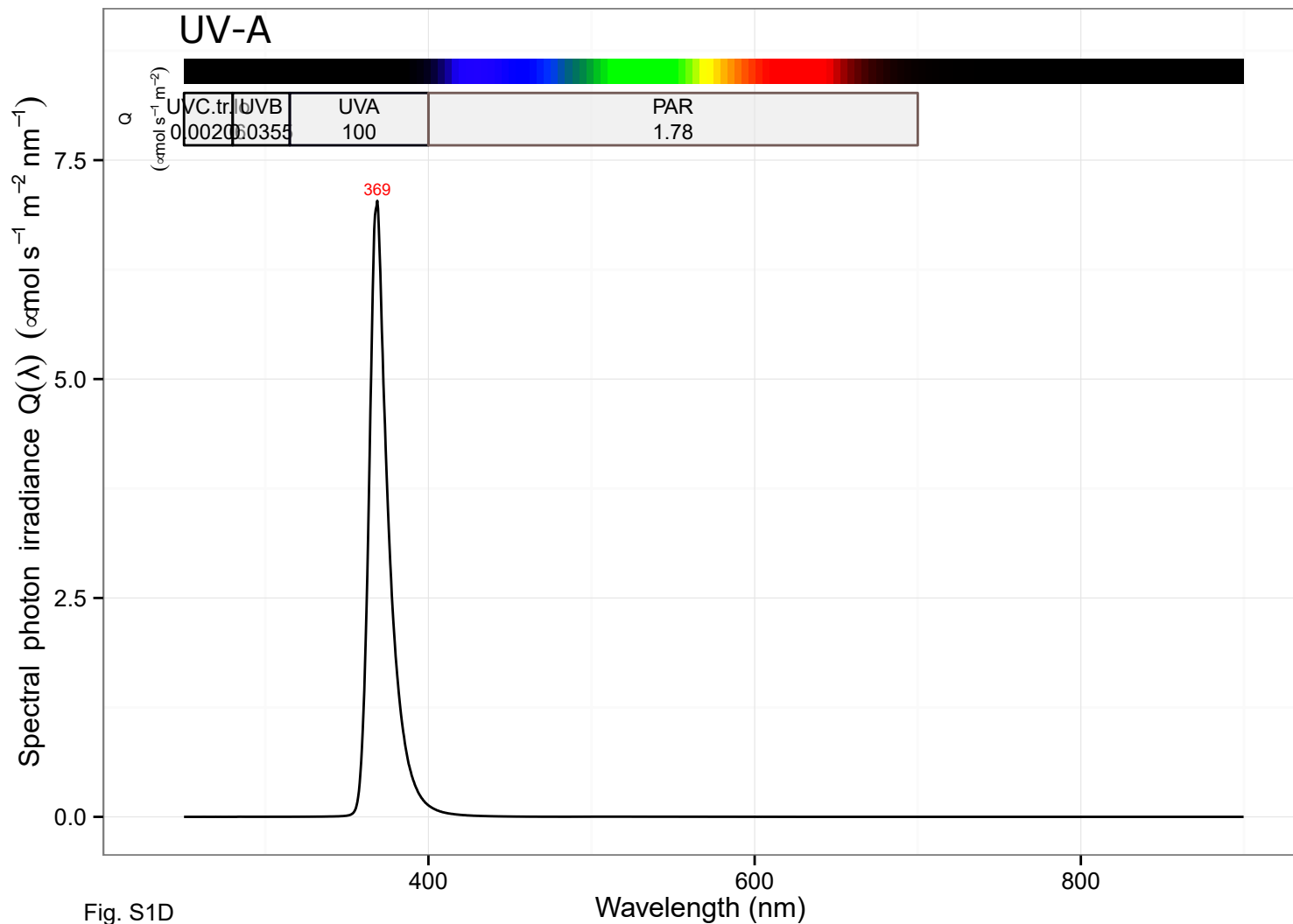

Fig. S1D

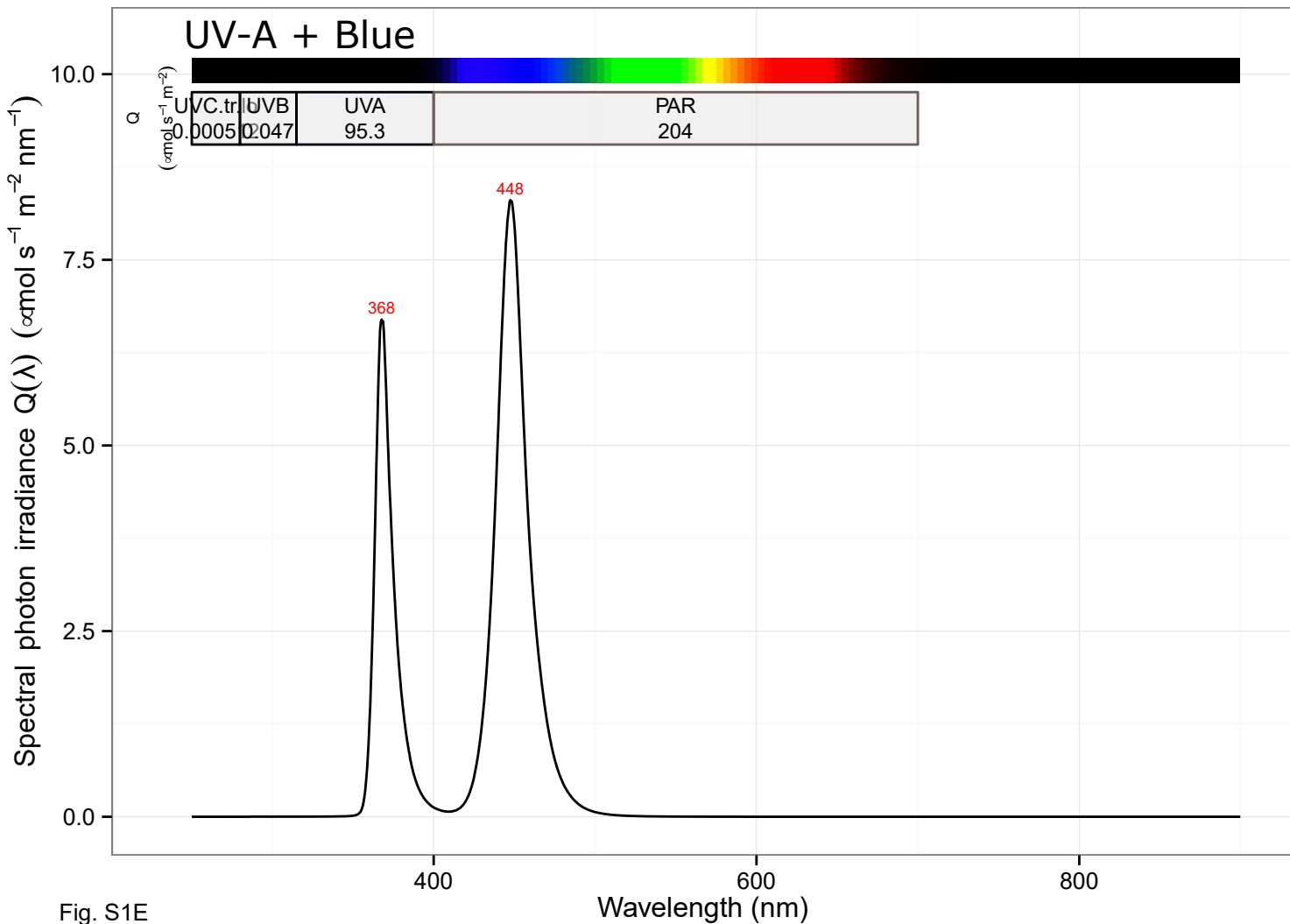

Fig. S1E

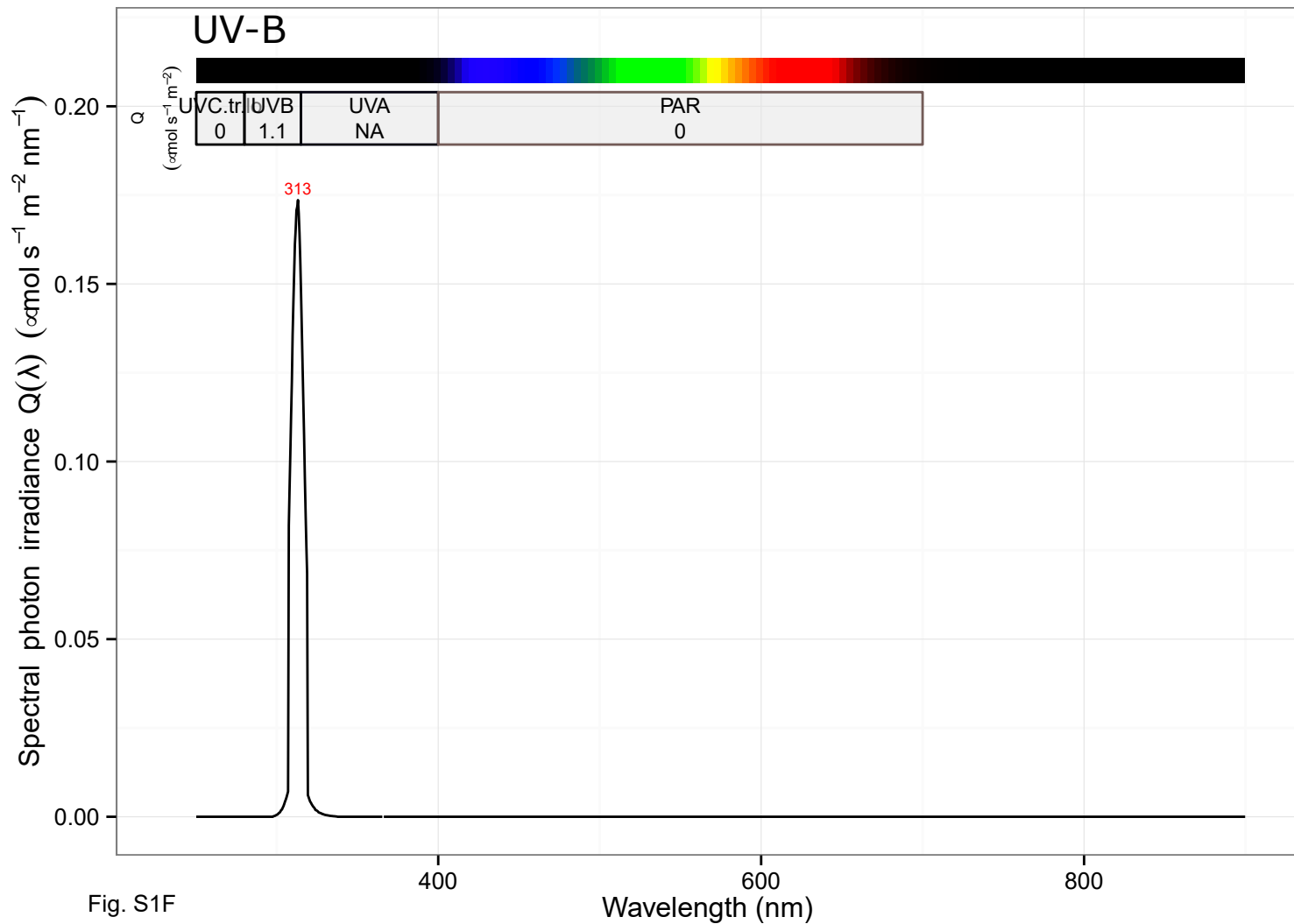

Fig. S1F

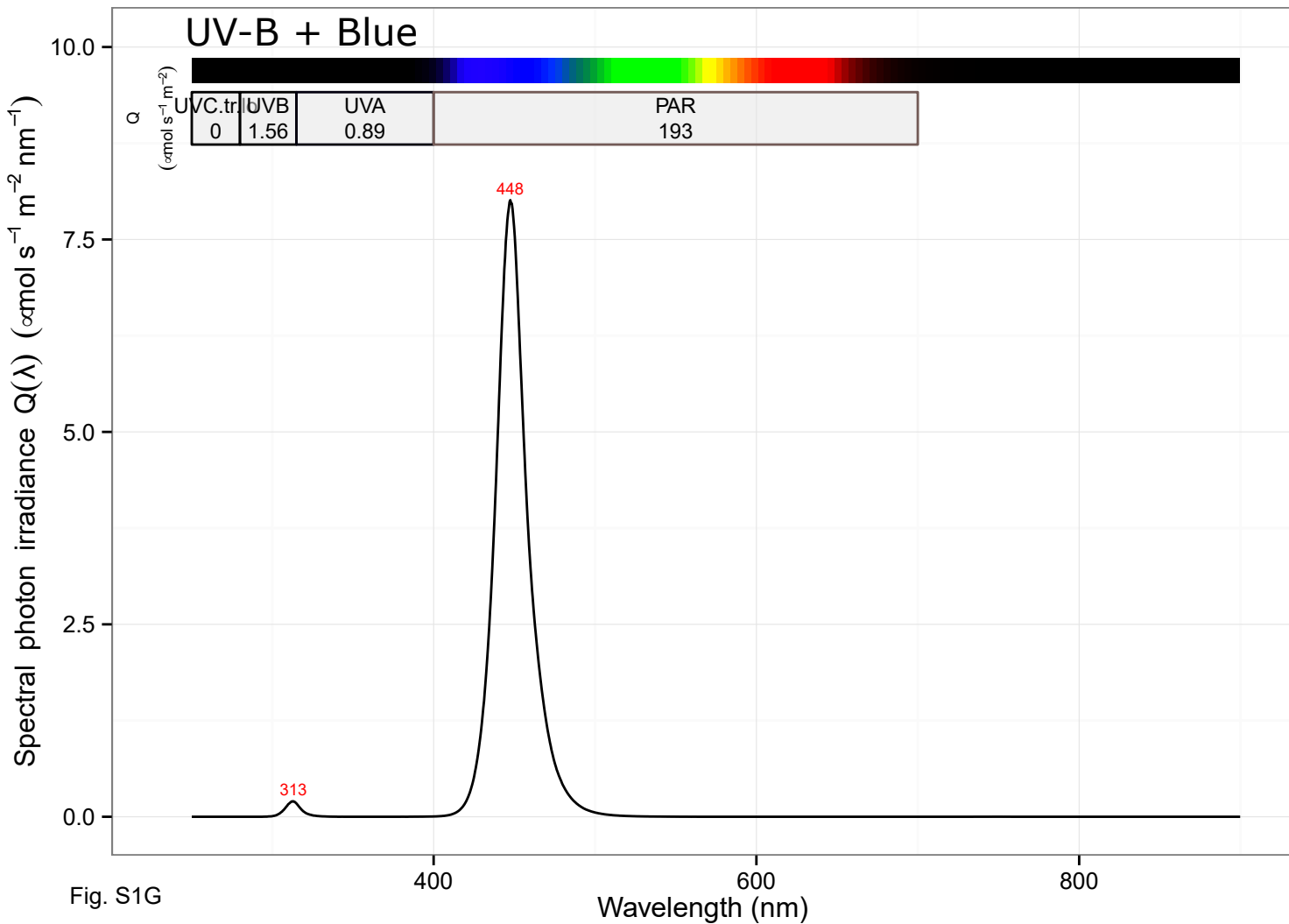

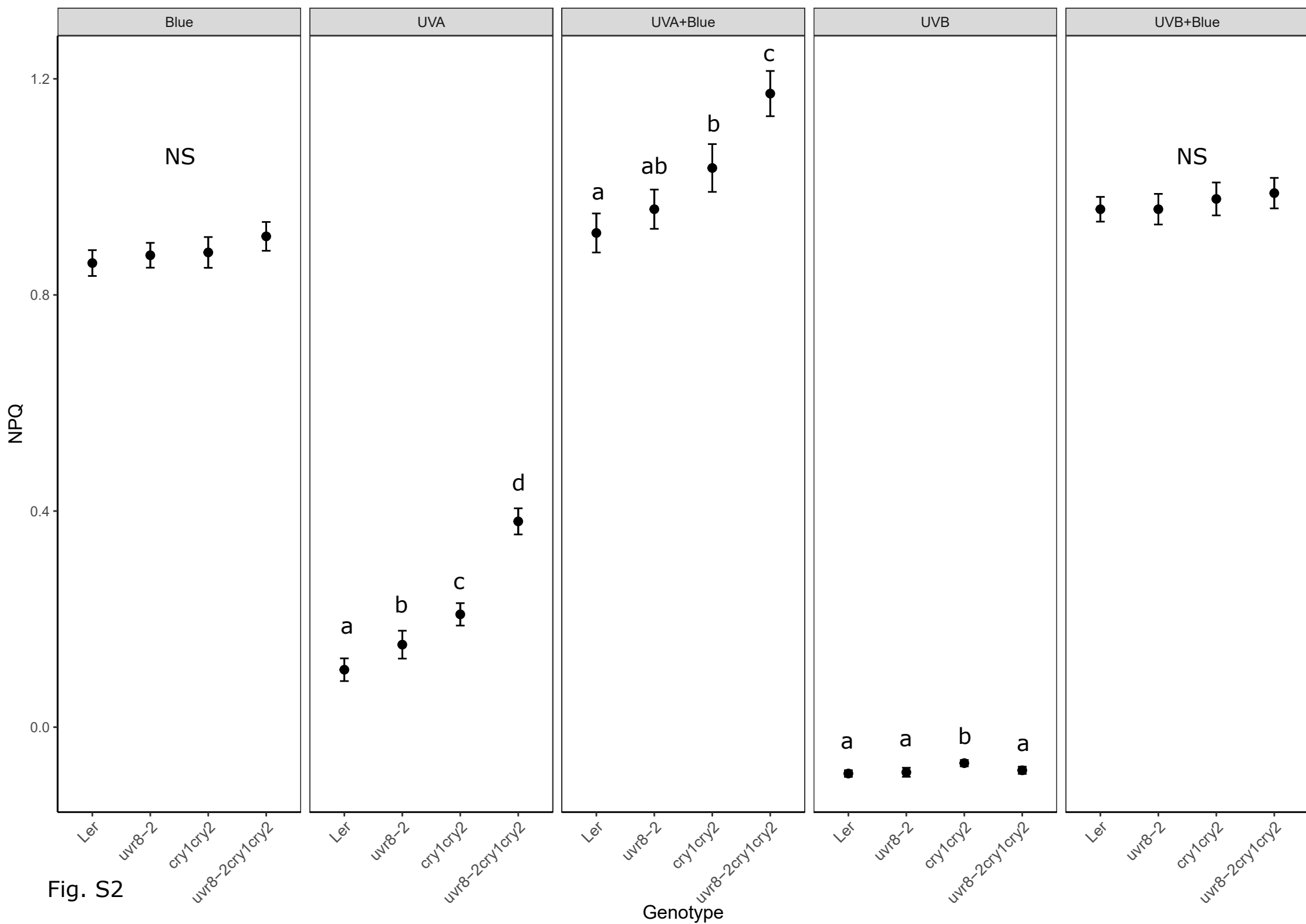

Fig. S2

Fig. S3

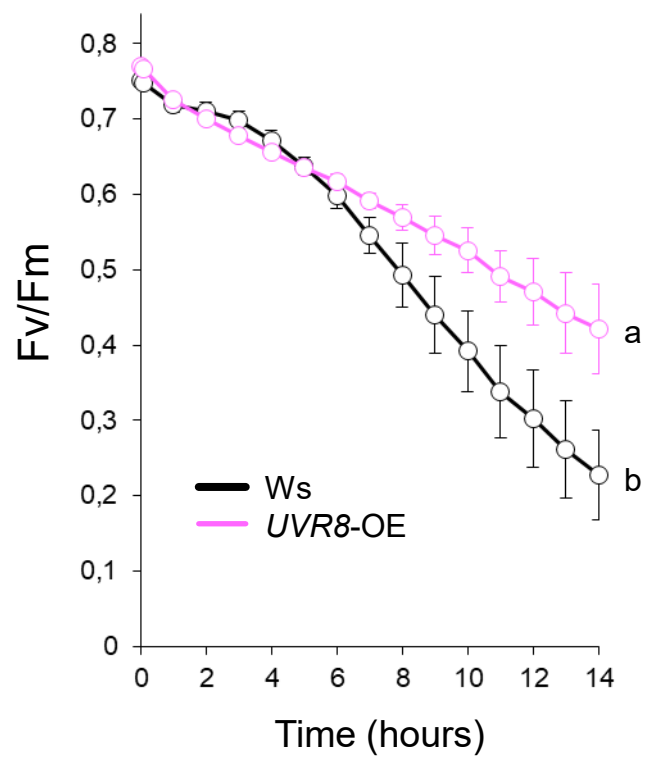

Fig. S4

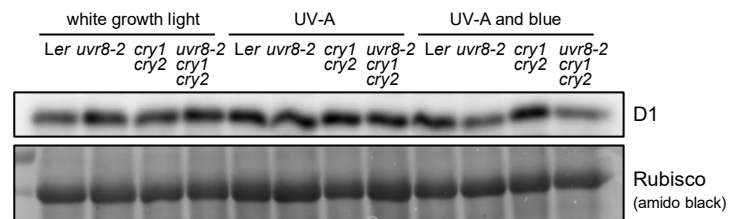

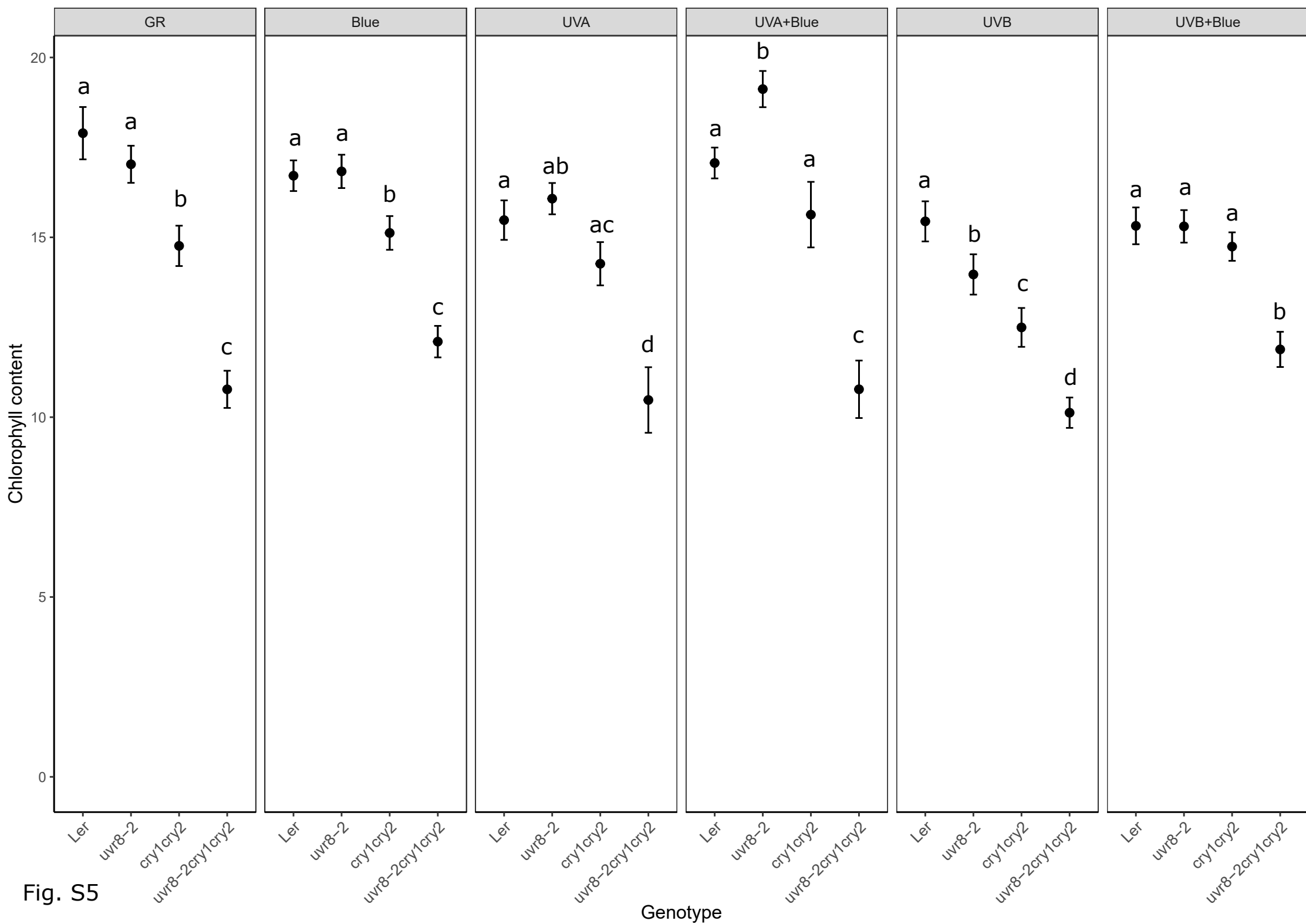

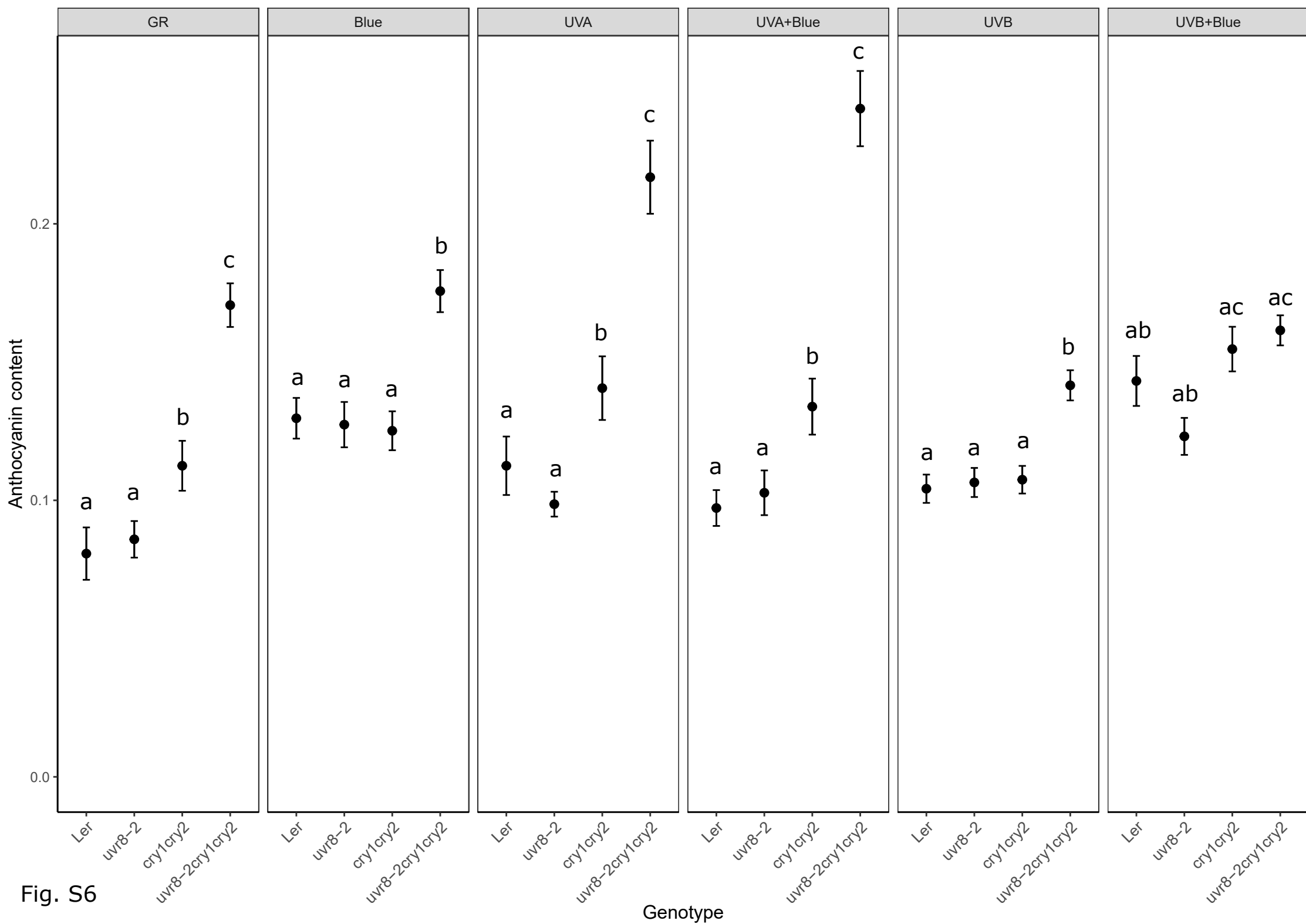

Fig. S6

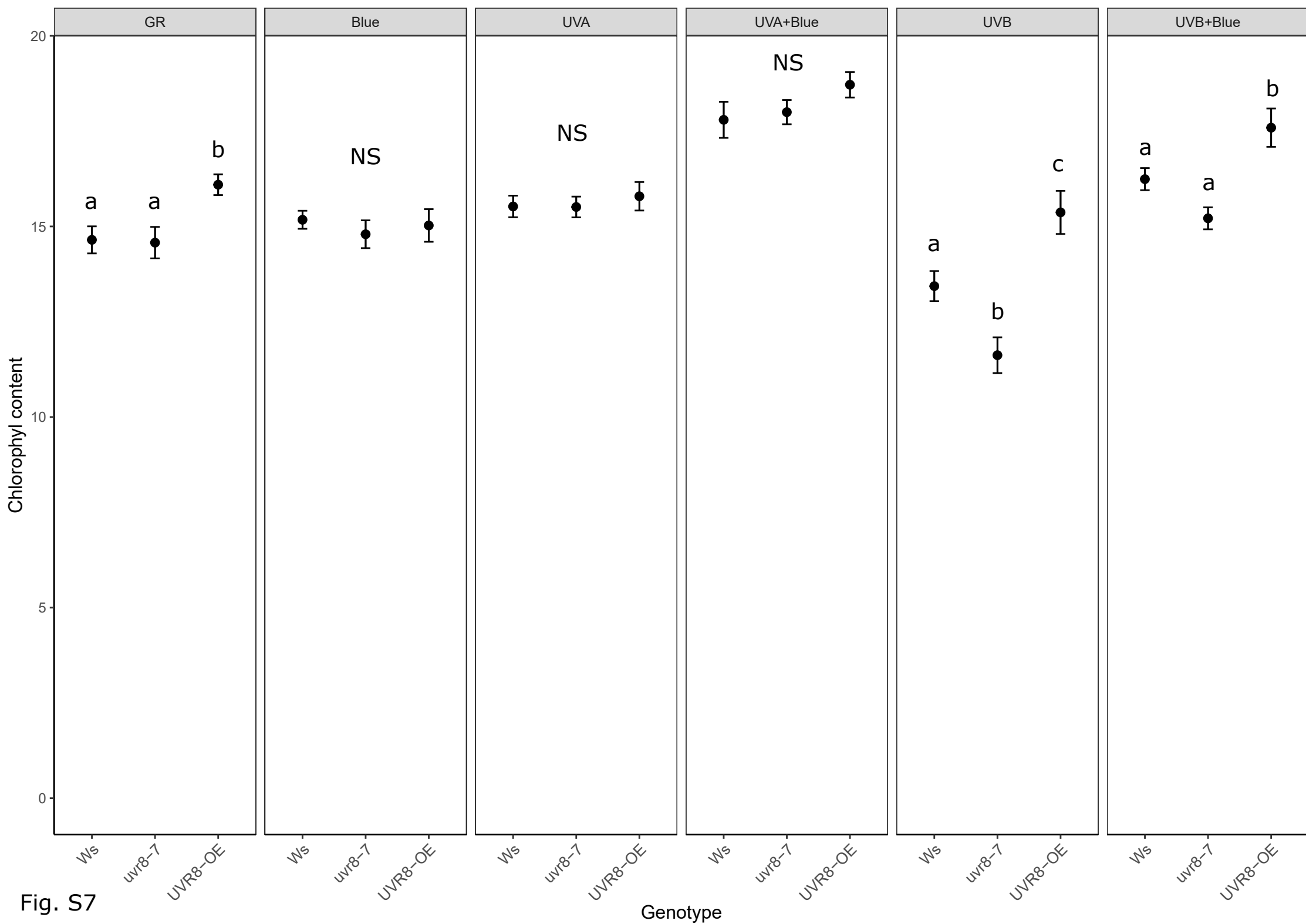

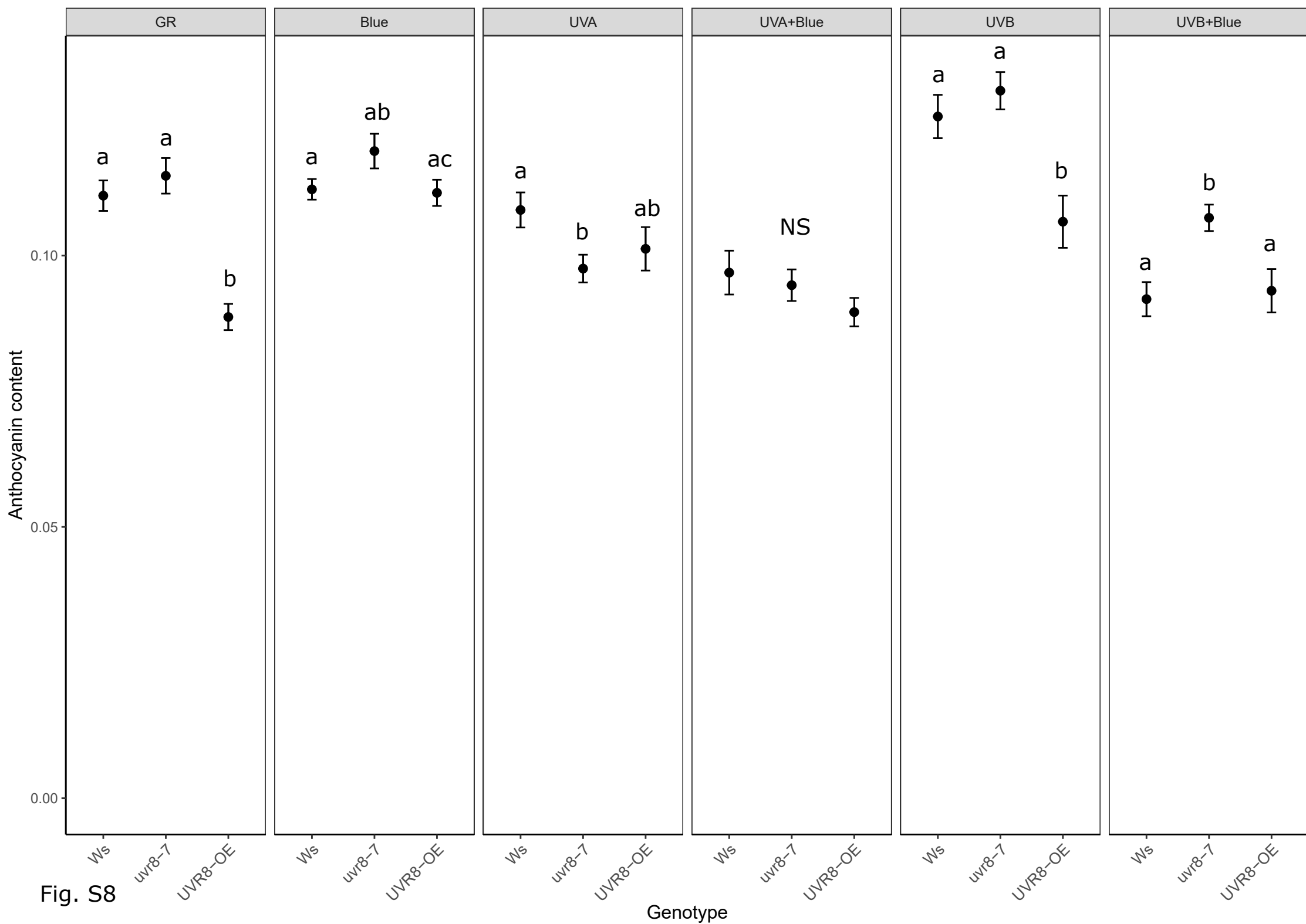

Supplement: Supplementary file 1 — Fig. S1. Spectral distribution and irradiance used in each light treatment. Fig. S2. NPQ measured after 30 minutes of light exposure. Fig. S3. Fv/Fm measured in leaf discs of Ws and UVR8‐OE exposed to methyl viologen. Significant differences (P< 0.05) between genotypes are denoted with different letters. The experiment was repeated three independent times. Fig. S4. D1 levels measured in Ler, uvr8‐2, cry1cry2 and uvr8‐2cry1cry2 exposed to UVA1 (365 nm 100 µmol m‐2 s‐1), UV‐A1 + Blue light, 450 nm 204 µmol m‐2 s‐1, or GR (plants grown in growth rooms in parallel with treated plants. The experiment was repeated two independent times. Fig. S5. Chlorophyll content estimated with Dualex in Ler background genotypes exposed to different light treatments for 20 h. Blue (Blue light, 450 nm 230 µmol m‐2 s‐1), UVA (UV‐A1, 365 nm 100 µmol m‐2 s‐1), UVB (UV‐B, 310 nm 1 µmol m‐2 s‐1), GR (plants grown in growth rooms in parallel with treated plants ‐ but not treated with UV or blue). The data points represent means of four independent biological repeats, and the error bars indicate the SE. In each experiment two leaves from 6 plants of each genotype were measured. In each experiment n = 6 plants of each genotype were measured. Significant differences (P< 0.05) between genotypes under each light treatment are denoted with different letters. Fig. S6. Anthocyanin content estimated with Dualex in Ler background genotypes exposed to different light treatments for 20 h. Blue (Blue light, 450 nm 230 µmol m‐2 s‐1), UVA (UV‐A1, 365 nm 100 µmol m‐2 s‐1), UVB (UV‐B, 310 nm 1 µmol m‐2 s‐1), GR (plants grown in growth rooms in parallel with treated plants ‐ but not treated with UV or blue). The data points represent means of four independent biological repeats, and the error bars indicate the SE. In each experiment two leaves from 6 plants of each genotype were measured. Significant differences (P< 0.05) between genotypes under each light treatment are denoted with different letters. Fig. S7. [file PCE-48-6321-s002.pdf]
